# Protein Complex Identification and quantitative complexome by CN-PAGE

**DOI:** 10.1038/s41598-019-47829-7

**Published:** 2019-08-08

**Authors:** Michal Gorka, Corné Swart, Beata Siemiatkowska, Silvia Martínez-Jaime, Aleksandra Skirycz, Sebastian Streb, Alexander Graf

**Affiliations:** 1 Max Planck Institute of Molecular Plant Physiology, Am Mühlenberg 1, 14476 Golm, Germany; 2 Celon Pharma SA., Ogrodowa 2 A, 05-092 Łomianki/Kiełpin, Poland; 3 Functional Genomics Center Zurich, ETH Zurich, Winterthurerstr. 190, 8057 Zürich, Switzerland; 4 Smak Academy, Pappelallee 45, 14469 Potsdam, Germany

**Keywords:** Plant sciences, Proteome, Protein-protein interaction networks

## Abstract

The majority of cellular processes are carried out by protein complexes. Various size fractionation methods have previously been combined with mass spectrometry to identify protein complexes. However, most of these approaches lack the quantitative information which is required to understand how changes of protein complex abundance and composition affect metabolic fluxes. In this paper we present a proof of concept approach to quantitatively study the complexome in the model plant *Arabidopsis thaliana* at the end of the day (ED) and the end of the night (EN). We show that size-fractionation of native protein complexes by Clear-Native-PAGE (CN-PAGE), coupled with mass spectrometry can be used to establish abundance profiles along the molecular weight gradient. Furthermore, by deconvoluting complex protein abundance profiles, we were able to drastically improve the clustering of protein profiles. To identify putative interaction partners, and ultimately protein complexes, our approach calculates the Euclidian distance between protein profile pairs. Acceptable threshold values are based on a cut-off that is optimized by a receiver-operator characteristic (ROC) curve analysis. Our approach shows low technical variation and can easily be adapted to study in the complexome in any biological system.

## Introduction

Protein function and activity are highly regulated within living cells. To become functional, proteins commonly depend on their interactions with other molecules. These molecules are known to include other proteins, which readily interact to form protein complexes. The majority of cellular processes are dependent on protein-protein interactions (PPI’s)^1^. An additional layer of complexity is added by the interaction of different protein complexes in so called super-complexes or metabolons^2^. The formation and rearrangement of super-complexes and metabolons has an influence on metabolic fluxes and helps plants to adapt metabolic pathways in response to environmental changes^3^. Therefore, global and quantitative analysis of protein complexes is required to understand how the organization of proteins into functional units is connected to the regulation of metabolic pathways.

Studying protein complexes is a technically challenging and time-consuming task. The most common experimental approaches used to identify protein complexes rely on the classical yeast two-hybrid (Y2H) system or affinity purification in combination with mass spectrometry (AP-MS). Each of these have advantages and limitations, especially concerning the sensitivity and specificity of the method, and its potential for scalability^4,5^. The Y2H system, although scalable to high throughput, is limited by the large number of false positive and false negative results that are generated^6,7^. Further, it often has a rather limited binary readout for a theoretical possible interaction, without discrimination of interaction stability, subcellular localization and subunit stoichiometry. AP-MS strategies often require modifications of the biological system, which not only alters the native structure of the protein by the introduction of a tag, but also the quantity as a result of overexpression. Furthermore, this method is labour intensive and limited to studying a small number of proteins in parallel^8,9^. Moreover, the ability to obtain quantitative data on protein complex abundance and composition from AP-MS experiments is very limited.

In an attempt to overcome the challenges of the aforementioned methods, researchers combined classical methods of protein fractionation, such as polyacrylamide gel electrophoresis (PAGE) or column based chromatography with quantitative mass spectrometry. One such method, 2-D PAGE remains an accessible method for separating and studying protein complexes. In contrast to the previously described approaches, it can provide quantitative data on hundreds of proteins. More recently, researchers have used one-dimensional fractionation of protein complexes by size-exclusion chromatography (SEC) or blue-native PAGE (BN) to obtain size-distribution profiles for up to 3400 proteins in a single experiment^10–12^. One-dimensional fractionation is not only less laborious than 2-D PAGE, but it also provides more reproducible protein separation at a high resolution.

To achieve the goal of studying protein complexes within the complexome comparatively, the currently used methods need to be extended to include quantitative and proteome-wide analyses of protein complexes. Here we propose a method for the isolation and identification of protein complexes using native-PAGE (NP) for the separation of protein complexes in their native state. Our approach works to overcome the limitations of other methods and provides high quality qualitative and quantitative data. To minimize technical variation, the samples from different biological replicates and conditions were processed in parallel, thereby minimizing technical variation. The importance of minimization of technical variation implementation has been also recently highlighted in complexome studies^13^.

We show that size-distribution profiles of proteins that participate in different complexes can be deconvoluted, in turn isolating peaks and improving the clustering of our data. Using the proteasome as a benchmark protein complex we show that our native PAGE analysis effectively separates and identifies the two proteasomal subunits with a high coverage of subunits. Furthermore, we analyse the protein complexome of *Arabidopsis thaliana* at end of day (ED) and end of night (EN). The highly reproducible nature of the data across biological replicates facilitates the identification of changes in protein complex abundance between ED and EN. This method is a step towards comparative analyses of protein complexes in different conditions or genotypes. Our approach can easily be adapted for any biological system.

## Results and Discussion

### Experimental design and data reproducibility

**Figure 1:**
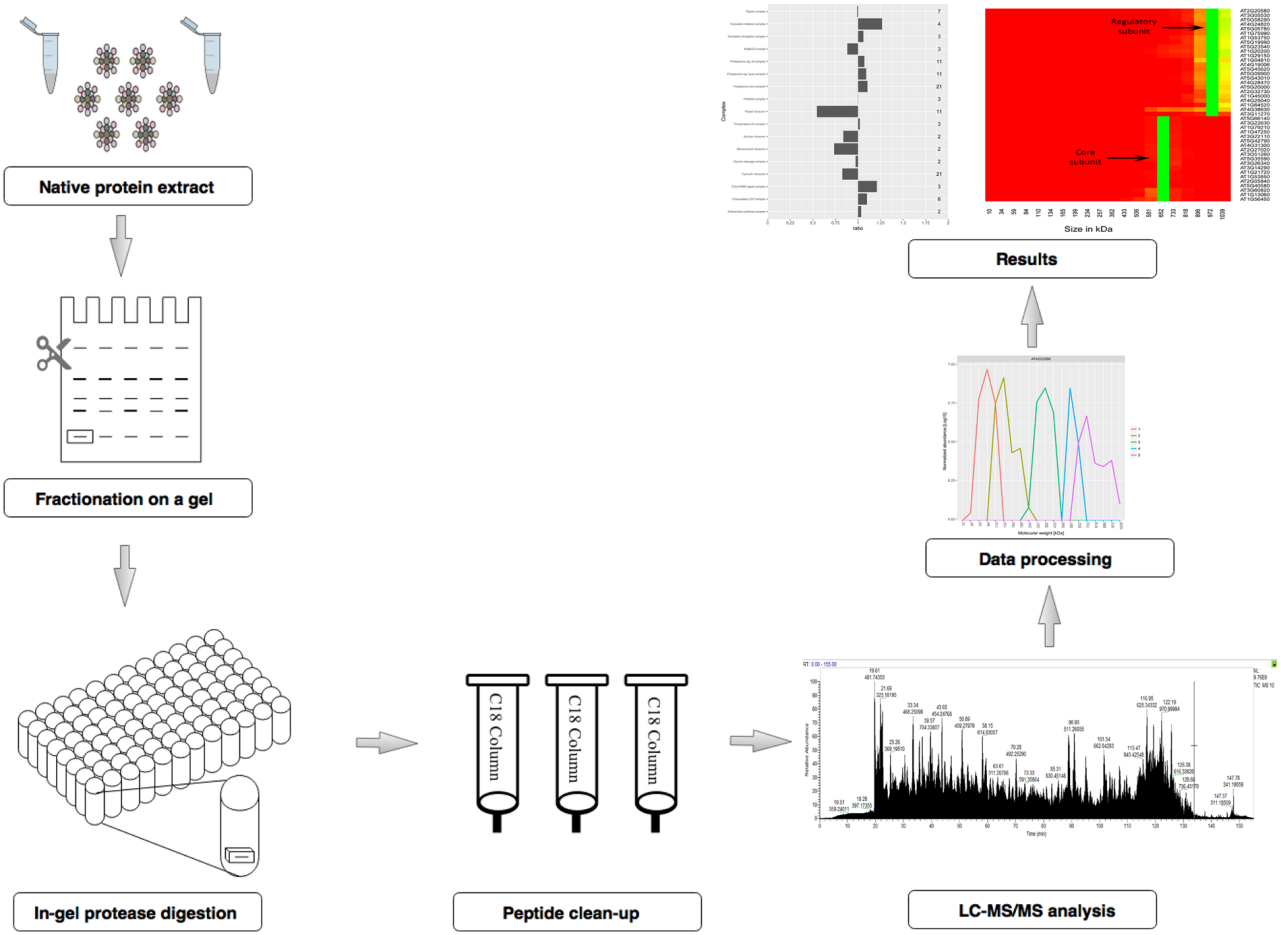
Visual representation of the experimental workflow used to study complexome changes. Schematic representation of the workflow used to study changes in the protein complexome. Protein extracts were prepared from fully expanded *Arabidopsis thaliana* leaves under native conditions and separated by native-PAGE. The proteins were subsequently digested using a modified in-gel digestion procedure, and the peptides desalted using C18 columns. The obtained peptides were analysed by LC-MS/MS and the raw spectral information exported to MaxQuant for protein identification and peak based label-free quantification. All downstream data analyses were performed using R statistical software.

LC-MS/MS based quantitative proteomics studies yield large and informative datasets, but are susceptible to large variation that negatively impacts statistical analyses. It is therefore highly advisable to safeguard against technical variation by developing rigorous and strict workflows^14^. Great care should be taken to select the appropriate methods that are used for protein complex extraction, fractionation and sample preparation for mass spectrometry, any or all of which could serve as the source of technical variation. With this in mind we established a workflow to allow processing of native protein extracts for the comparative analysis of the plant complexome at different diurnal time points. In this approach the isolated protein extracts are separated by native PAGE fractionation, followed by a modified in gel digestion procedure and analysis by tandem mass spectrometry (Fig. 1). Native-PAGE was chosen for protein separation over the commonly employed SEC because it provides a simple, efficient and cost-effective solution for protein fractionation^10,12,15,16^. It also allows for an experimental design where multiple replicates of the samples can be prepared and run in parallel on a single gel. This in turn reduces the risk of introducing additional technical variation that usually stems from sample preparation and separation.

**Figure 2:**
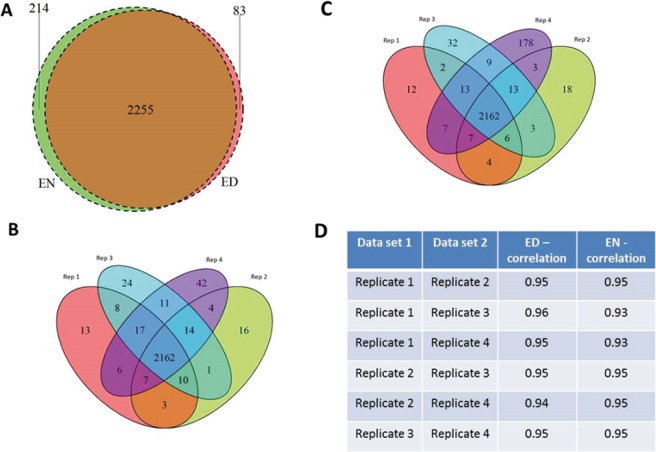
Evaluation of protein identification and reproducibility within the datasets. Label free quantification of the raw spectral information was performed in MaxQuant to produce protein datasets at the ED and EN. (**A**) The overlap between all identified proteins at the ED and EN was represented as a Venn diagram. (**B**) As a measure of reproducibility the overlap of the 4 biological replicates at the (**B**) ED and (**C**) EN was calculated and visualised as a Venn diagram. (**D**) A Pearson’s correlation matrix was performed as an additional measure of reproducibility at the protein level. Correlation factors were calculated for proteins in a pairwise manner across the entire matrix for each fraction of each biological replicate versus another.

As a proof of concept study, we decided to analyse *Arabidopsis thaliana* Col-0 plants harvested at the end of the light period (ED), when plants are photosynthetically active, and the end of the night (EN), when plant growth relies on internal stored carbon reserves. Native proteins and intact protein complexes were extracted from four biological replicates at 4 °C in a detergent-free buffer and separated using 1D native PAGE. The native gel prepared for this study showed highly reproducible protein separation for all lanes. This was essential for producing 20 fractions for each of the eight biological samples by cutting across the full width of the gel (Supplemental Fig. 1). To minimize technical variation and contamination, sample fractions were prepared for mass spectrometry analysis using HiT-Gel, a recently established, high-throughput in-gel digestion method^17^. The raw spectral data of the 160 sample fractions was processed with MaxQuant. We identified 2338 proteins based on unique peptides at ED and 2469 at EN with and overlap of 88.3% between the two conditions (Fig. 2). A high degree of overlap is a prerequisite for accurate comparison of the changes in the complexome at the ED and the EN. The overlap between the biological replicates at ED and EN was represented as a Venn diagram (Fig. 2) with an overlap of 92,5% (ED) and 87,6% (EN). We calculated the coefficient of variation (CV) using the relative protein abundances of all identified proteins in the four biological replicates at both time points. The result was represented as a histogram with a normal curve (Supplemental Fig. 2A,B). We also used the relative protein abundances for all identified proteins as input for a Pearson’s correlation analysis between all possible pairs of biological replicates of the same condition (Fig. 2D). In each of the pairwise comparisons we a found a correlation higher than 0.9. We can therefore conclude that our experimental workflow gives rise to reproducible proteomics data, with high overlaps in identified proteins and low sample to sample variation. To determine if high reproducibility between our experiments is not due to horizontal carryover, we run an additional experiment. The same amount of protein extract (300 µg) were run on two, central lanes of native-PAGE gel. Ten randomly selected gel fractions were excised, processed and analysed by LC-MS/MS (Supplemental Fig. 2C). In total 20 proteins were identified what suggest minimal horizontal carryover between gel lanes. Majority of identified proteins were annotated by single peptide (Supplemental Fig. 2D).

### Protein profiling and deconvolution of profiles

The main aim of our study was to establish an effective method for the comparative analysis of protein complexes. Following protein identification and quantification, the migration profiles of all proteins along the molecular weight gradient were calculated to allow further data analyses such as clustering, protein complex identification and statistical analysis of changes in protein complex abundance between ED and EN.

**Figure 3:**
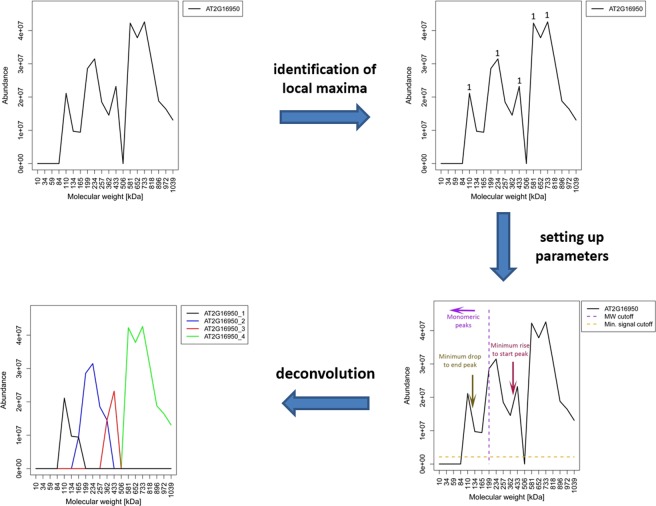
Schematic representation of the deconvolution of a protein profile. A single protein from the ED experiment was selected as an example to illustrate the procedure wherein a protein profile is deconvoluted. In this case the elution profile of the selected protein could be simplified into four distinct peaks. In the first step the algorithm identifies the local maxima in the complex elution profile. Once the local maxima have been defined, a strict filtering process is performed to identify individual peaks that are most likely hidden within the complex profile. At this stage the newly identified peaks are compared to the local maxima, and deconvoluted peaks are generated where a clear overlap is observed.

At the core of our data processing pipeline is the statistical software R. Our data analysis pipeline starts with the reconstruction of protein migration profiles by combining the neighbouring fractions as excised form the gel. In the next step, we identified the local maxima (peaks) along the molecular weight gradient for each protein using the turnpoints function (pastecs package) in R (Fig. 3). The local maxima were filtered to remove maxima below 20% of the highest relative abundance along the protein profile.

Next, we separated the apparent masses for all identified protein peaks from their monomeric masses, which we obtained from the TAIR10 database^18^. This provided us with an overview of the oligomerization state (R_app_) of all the protein peaks identified in our experiments. The distribution of the apparent masses of identified proteins was visually summarized using scatterplots (Supplemental Fig. 3). We applied a two-fold monomeric weight cut-off to our data, because it was previously shown to be a good threshold for the identification of proteins in oligomeric assemblies^10,15^. The majority of identified proteins (89%) in our data peaked in fractions corresponding to size ranges larger than their monomeric mass. Fewer than 11% of the peaks were identified in a monomeric range and proteins experienced minimal degradation as indicated by the small number of peaks with a R_app_ of less than 0.5. This data shows that most of the proteins are found in complexes. As an additional proof of protein complexes in the dataset we used a histogram to summarize the distribution of monomeric masses in comparison to the observed size ranges of each local maximum (Supplemental Fig. 4). The histogram indicated that the monomeric masses were mostly observed in a narrow and lower mass range window than those of the apparent masses. This again confirms that protein complexes are captured in our dataset.

Subsequently, each of the protein chromatograms was deconvoluted into distinct peaks that represent independent homo- or heteromeric states of protein oligomerization. In the event of a protein interaction, proteins that interact to form an assembly will also share a common peak. Because of the heterogenous composition of protein complexes, it is not expected that all possible interactors will show identical protein elution profiles. Interaction partners might rather share one peak but have an overall very distant profile. Consequently, complex protein profiles are problematic in clustering approaches that rely on the Euclidean distance between whole profiles. Following deconvolution each peak can be treated as a separate entity independent of further peaks in the original profile (Fig. 3). While profile deconvolution has been successfully implemented in SEC-based analyses of mammalian protein complexes, it was to our knowledge not performed in omics-scale studies in plants and PAGE based approaches

We used a set of 4 parameters to deconvolute the protein abundance profiles into isolated peaks. Briefly, the profile was analysed by moving from fraction to fraction and starting at the lowest molecular weight. At each position, a decision tree was used. Only relative protein abundances of more than 5% of the maximum abundance in the total profile were considered to be part of a deconvoluted peak (minimal considered signal). If the abundance of a fraction exceeds the abundance of the previous one the profile is considered on the rise towards a peak and the next fractions is analysed. If the abundance decreases to < 70% of the maximal abundance in the current peak, the script considers that the profile is entering the downward slope and the peak fraction is fixed. A peak is defined as finished when the signal crosses the minimal considered signal or the relative abundance in a following fraction is increased by > 30% in comparison to the previous one. Finally, the putative peak has to fulfil two more criteria to be accepted by the deconvolution script: Firstly, the recorded peak fraction has to be annotated as local maxima (see above), and secondly, the relative abundance at the peak position has to be > 20% of the maximum abundance in the total profile. Next, we removed the deconvoluted peaks representing the monomeric state by comparing the size of each peak with the monomeric molecular weight of the respective protein according to the TAIR10 database.

**Figure 4:**
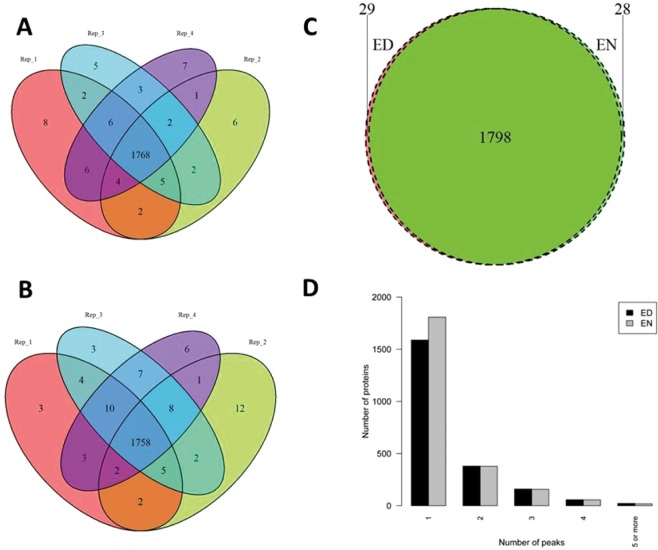
Evaluation of protein complex identification between the ED and EN. The overlap between protein complexes identified in each of the biological replicates at the (**A**) ED and (**B**) EN were calculated and visualised as Venn diagrams. (**C**) A Venn diagram was also produced to show the overlap between the total number of protein in complexes identified at the ED and the EN. (**D**) Proteins that participate in complex formation should have at least one peak in addition to their monomeric peak. To illustrate the likelihood of complex formation, the number of peaks produced by each protein was visualised as a barplot.

Recent approaches to study the complexome in plants have been unable to overcome the challenge presented by complex protein profiles in native fractionation experiments^10,15,16^. To circumvent the problem of peak shape and multiple peaks per protein profile, researchers focused their analysis on the position of the maximal protein abundance in a profile and considered proteins with similar maxima as putative interaction partners. This solution does offer insight into protein complex formation, but it excludes the possibility of identifying co-eluting protein complexes in the remaining fractions. It has previously been reported that the deconvolution of the elution profiles are a possible solution and we applied our own method of deconvolution to address the problem^12^. Using a two-fold molecular weight cut-off to exclude deconvoluted peaks representing monomeric proteins, we found 1768 (ED) and 1758 (EN) proteins in a size range indicating protein complex formation (Fig. 4A–C) with an overlap of 1798 proteins between ED and EN. The majority (approximately 74%) of proteins only show a single peak indicative of complex formation while approximately 16% have two peaks, 7% 3 and 2% 4 peaks (Fig. 4D). Five peaks were only observed in approximately less than 1% of the proteins. Consequently, most proteins are detected in only one protein complex in our native PAGE analysis.

### Impact of profile deconvolution on data analysis

**Figure 5:**
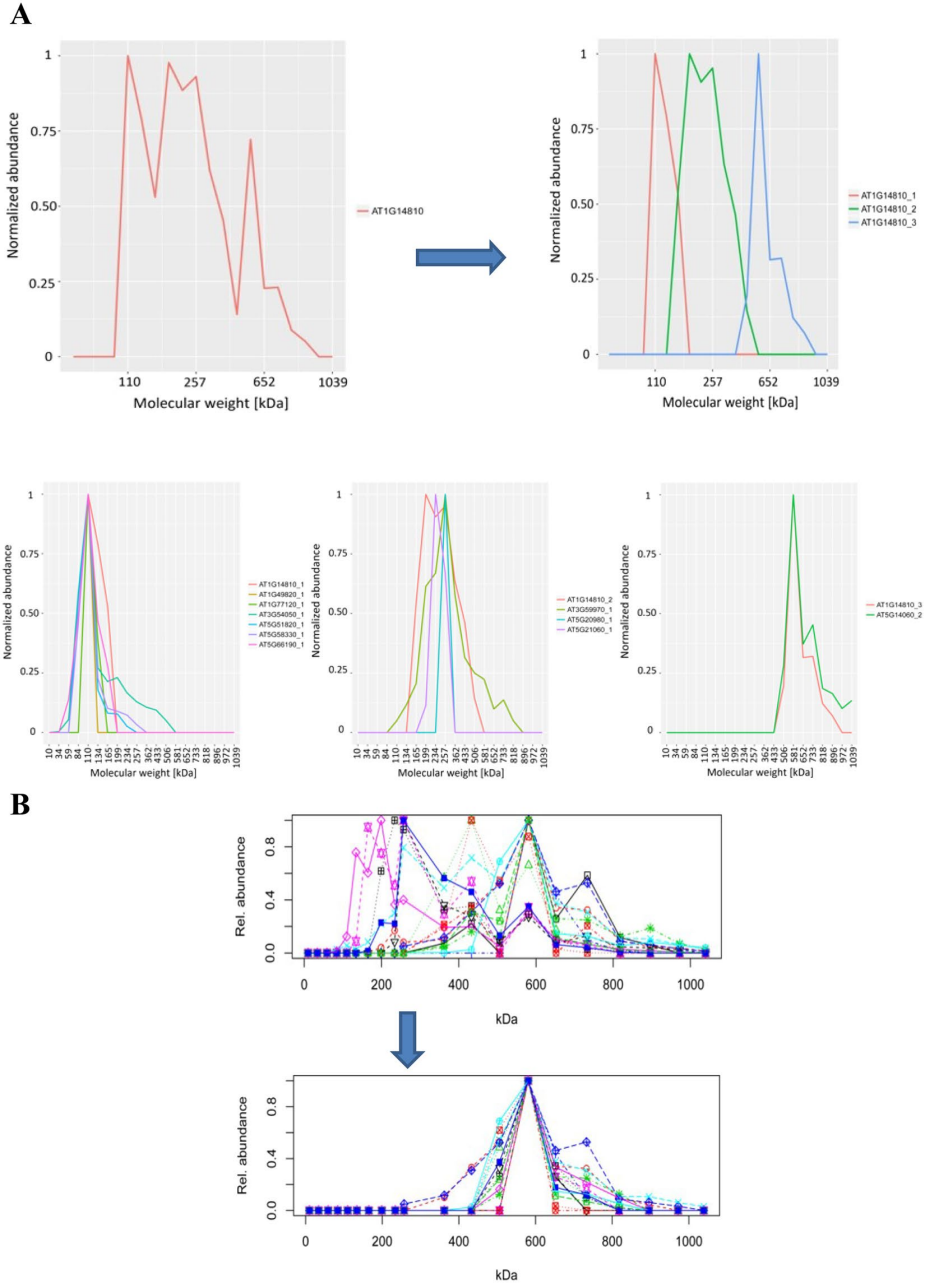
The importance of protein profile deconvolution for protein complex identification. The migration profile of aspartate semialdehyde dehydrogenase was deconvoluted into three distinct peaks. (**A**) Each of the deconvoluted peaks was found in a different cluster following a hierarchical clustering analysis. The profiles of proteins found in the same cluster, which also function in the same cellular process as aspartate semialdehyde dehydrogenase were visually represented. From the left: oxidation-reduction process (AT1G14810_1), methionine biosynthetic process (AT1G14810_2), lysine biosynthetic process via diaminopimelate (AT1G14810_3). (**B**) To determine the impact of deconvolution on very complex protein profiles, an elution profile of a number of proteins from the pentose-phosphate pathway is shown. In the upper panel no deconvolution is applied, and as a result hierarchical clustering could not be used to identify the proteins as being in a complex. In the lower panel deconvolution was performed, which provided clearly defined peaks that could be used in the same hierarchical clustering approach to identify a complex of 16 proteins.

To demonstrate the value of deconvolution we used the elution profile of aspartate semialdehyde dehydrogenase (AT1G14810) as an example (Fig. 5A). After deconvolution of the native size distribution profile we obtained three distinct peaks. Next, all deconvoluted peaks were clustered based on their Euclidian distance. The three peaks of aspartate semialdehyde dehydrogenase were identified in three independent clusters. We further analysed the clusters using GO annotations^19^ to identify functionally related proteins. In each of the clusters functionally related proteins were identified that could potentially form a complex with aspartate semialdehyde dehydrogenase (Fig. 5A). The putative interaction partners are unique to each cluster. We propose that aspartate semialdehyde dehydrogenase forms different oligomeric complexes in different size ranges. Identification of these clusters was not possible using the original and complex protein profile of aspartate semialdehyde dehydrogenase. This approach can also be used to effectively identify larger complexes with multiple subunits (Fig. 5B). It is however limited by the complexity of the data, and is not intended to unambiguously predict new interactions. Nevertheless, the data might be useful for validating predicted interactions or metabolon formation in a specific metabolic pathway. Furthermore, it could also aid in confirming the presence of known protein partners in a pathway or suggest novel putative interactors to guide future protein interaction studies on smaller scale.

The majority of proteins in the datasets were found to exist as protein assemblies (Fig. 4, Supplemental Figs 3 and 4). As these assemblies should be stable under native conditions, the subunits of these protein complexes should remain assembled during separation and produce comparable migration profiles. We decided to calculate the Euclidean distance between all possible pairs of deconvoluted peaks as a measure of similarity. To determine a Euclidean distance cut-off that separates predictions of interacting from non-interacting protein pairs, analysis of receiver operating characteristic (ROC) curve was performed. ROC curves require a dataset of true positive and true negative protein pairs (TP and TN). The TP dataset was created using Arabidopsis protein complexes annotated in the TAIR GO category “protein complex” (GO:0043234). The TN dataset consists of protein pairs with one protein being part of the TP set and the second randomly selected from the remaining dataset. ROC curves were generated for the ED and EN data using both, the original and deconvoluted protein profiles (Supplemental Fig. 5). Each point in a ROC curve represents the true positive rate (TPR), also called sensitivity, and false positive rate (FPR), or 1-specificity, within the protein pairs considered interacting at a specific Euclidean distance cut-off. We observed a steep rise of our ROC curves with a strong deviation from the line of no-discrimination. This shows that as the Euclidean distance cut-off is gradually increased, true positive interactions are discovered faster than false positive interactions (Supplemental Fig. 5). Moreover, ROC curves calculated based on deconvoluted protein profiles showed a slightly better curve indicating that deconvolution contributed to an improved specificity and sensitivity. Our ROC curves also indicated a higher sensitivity and specificity than previously reported for similar studies, supporting the quality of the generated datasets^12,20^.

### Reconstruction of biologically relevant pathways

In order to evaluate the quality of our dataset, we selected proteins from biologically relevant pathways and reconstructed the protein complexes using deconvoluted peaks.

**Figure 6:**
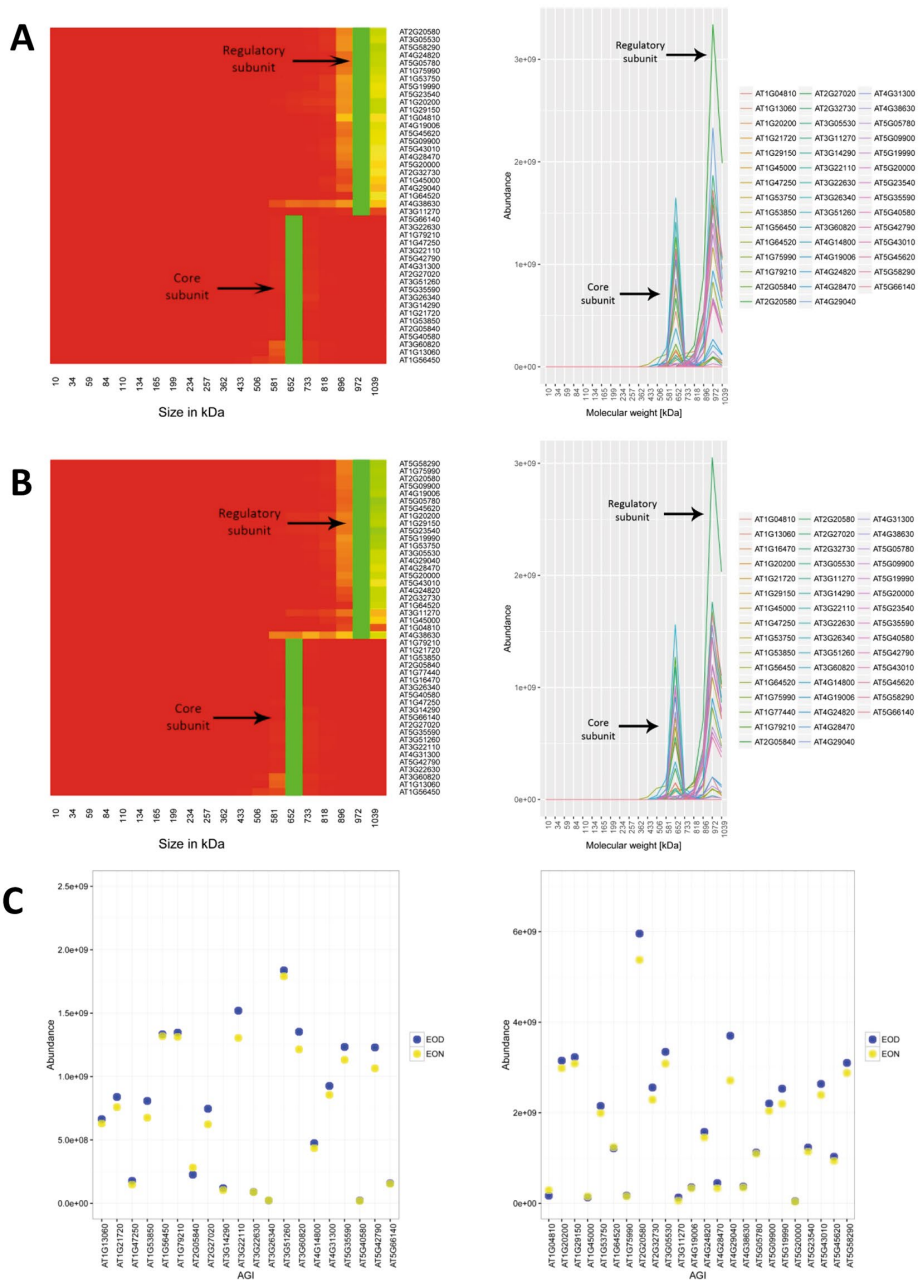
Comigration of the proteasomal subunit protein profiles. The protein profiles of the subunits comprising the proteasome complex were investigated at the (**A**) ED and (**B**) EN. Heatmaps shown represent the deconvoluted protein elution profiles of the proteins that belong to the regulatory and core subunits of the proteasome complex. The protein profiles were normalized to the maximum value. Green areas indicate the fraction where the highest abundance for a protein was observed. Comigration was observed for the constituents of the regulatory and core subunits of the proteasome complex. To the right of the heatmaps the protein elution profiles used to generate the respective heatmaps are shown. (**C**) A comparison of the protein abundance of the proteasomal proteins was performed at the ED and the EN. The total abundance for each of the proteins belonging to the core (left) and regulatory (right) subunits of the proteasome was compared between the two time points. The abundance of each of the proteins was summed across the fractions and are represented as yellow and blue circles for the ED and EN, respectively.

The first protein complex, the proteasome, is also the reference complex of choice to benchmark the quality of the dataset in other studies^10,15,16^. This complex is present in all eukaryotic cells and responsible for the degradation of proteins in the cytosol. The proteasome is comprised of a catalytic 20S core particle (CP) and a 19S regulatory subunit (RP)^21^. Represented visually as a heatmap, we were able to detect both the CP and the RP in easy to discern clusters (Fig. 6). The core subunit was also identified in a smaller size range than the regulatory subunit which holds true to the literature^22^. However, we were unable to detect the fully assembled proteasome. This was to be expected, as the fully assembled proteasome of *A. thaliana* has a molecular weight of approximately 2MDa and cannot be effectively captured with a native PAGE approach.

Based on the annotations in the KEGG database the CP should be present as a heteromeric assembly of 23 α- and β-subunits while the RP consists of 32 proteins^23^. Using a SEC-approach to study protein complexes in Arabidopsis Aryal *et al*.^10^ recovered 16 (70%) proteins of the CP and 3 proteins (9.3%) of the RP. We identified 20 (87%) and 22 (95%) co-migrating proteins of the CP at the ED and the EN, respectively (Fig. 6). For the RP were able to detect 24 (75%) co-migrating proteins at ED and EN (Fig. 6). This analysis shows, that our approach is highly reproducible and provides an improved coverage of the proteasome compared to previous studies^10,16^. We also extended this analysis to compare the relative abundances of the proteins that form the subunits of the proteasome at the ED and the EN (Fig. 6). The distribution of the protein ratios was observed to be stable indicating reproducible measurements at both of the time points.

As a second analysis, we intended to investigate the oligomerization states of specific enzymes in a well-known metabolic pathway. We selected the tricarboxylic acid (TCA) cycle to this end. The TCA cycle is present in the mitochondria and drives cellular respiration under oxidative conditions. It has been postulated that enzymes in the TCA cycle are able to form supramolecular complexes with weak and transient interactions^24,25^.

**Figure 7:**
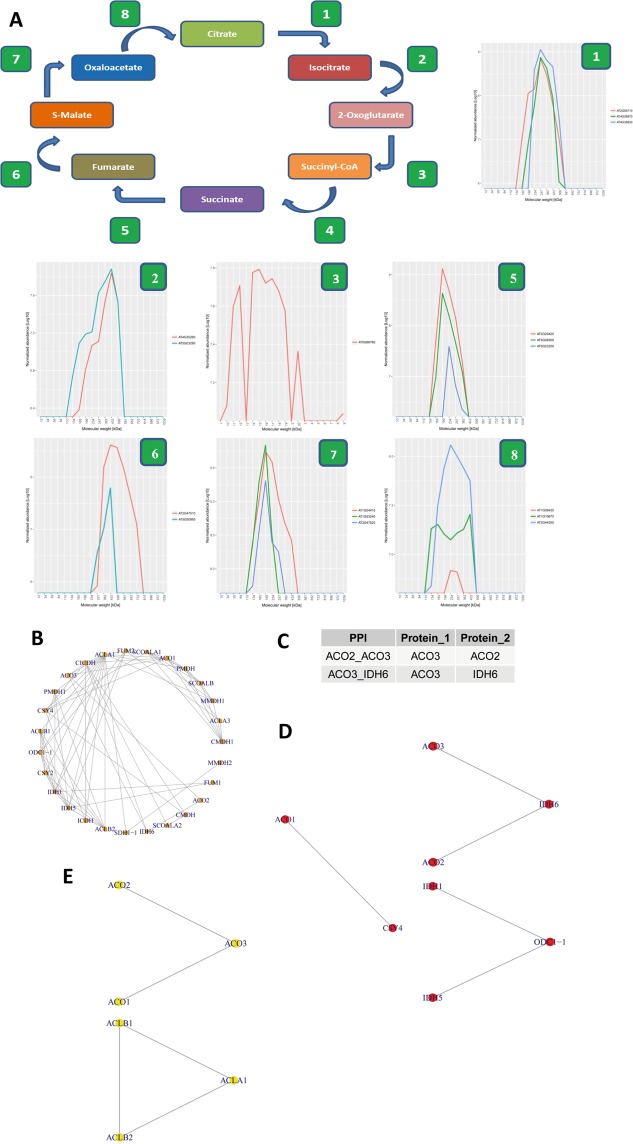
Using the TCA cycle as a scaffold to benchmark the identification of protein complexes. Using the KEGG database as a reference, a flow diagram of the TCA cycle was produced. In this diagram the enzymes that catalyse the individual substrate conversion reactions have been annotated as nodes. This flow diagram served as a scaffold wherein a comparison of the nodes was made to the ED dataset. It was possible to verify nearly all of the conversion reactions, with the exception of succinyl-CoA to to succinate. The enzymes involved in this step were not detected in any of the experimental replicates. Subsequently, the protein profiles of all detected enzymes at each individual node has been combined to visually illustrate power of our method to detect protein protein interactions. (**B**) Graphical overview of the PPI interaction network predicted for the TCA cycle using the ED data set. (**C**) The overlap between PPIs detected in the ED dataset and those identified by Zhang and colleagues are shown26. (**D**) Interactions identified between enzymes that catalyse consecutive reactions in the TCA cycle which were not reported by Zhang and colleagues26 (**E**) Predicted protein interactions within the enzyme protein complexes of aconitase (left) and ATP citrate lyase (right). Predicted interactions were observed using a Euclidean distance cutoff as predetermined using a ROC curve analysis (10% FDR). Enzymes and their isoforms are represented by nodes, while the edges connecting them show potential interactions.

To verify this theory, we applied our method to investigate 63 enzymes from the KEGG database that are involved in the reactions of the TCA cycle and the reverse TCA cycle (Fig. 7A). Of these 63 assigned proteins, we were able to detect 40 (63.5%). Nearly all of the detected enzymes had an oligomerization state (R_app_) of more than two, indicating complex formation (Supplemental Table 1). To discern which of the enzymes might interact with each other we selected the enzymes that are involved in the eight main reactions in the pathway and compared their elution profiles. In nearly all catalytic steps we could observe that the enzymes had very similar elution profiles across the size gradient, suggesting that they could form a protein complex (Fig. 7B).

Another important method of metabolic regulation depends on the micro-compartmentation of enzymes also known as metabolon formation^26–28^. Recently Zhang and coworkers showed the channeling of substaretes in a TCA cycle interactome, and revealed the interactome itself^29^. Using this PPI data as a reference we performed an analysis of protein complexes at the ED. In order to identify potential PPIs, in our CN-PAGE analysis, we calculated the Euclidean distance between the deconvoluted peaks of all TCA cycle related enzymes. Using our ROC curve analyses we set a Euclidean distance threshold based on a 10% FDR cut-off. If the calculated Euclidean distance between two deconvoluted peaks was smaller than the threshold value, we considered the protein pair to be interacting. We identified 27 TCA cycle related enzymes with a network of 74 interactions (Fig. 7B). The interaction network reported by Zhang *et al*. consists of 158 interactions between the 38 proteins annotated in the TCA cycle^29^. Comparing the two studies, only 11 proteins were found to overlap. These proteins produced 14 interactions in the study by Zhang *et al*. Of these 14 PPIs, only two (ACO2|ACO3 and ACO3|IDH6) were also observed in our analysis of interactions between TCA cycle enzymes^29^ (Fig. 7C).

While the study of Zhang and co-workers reported the existence of metabolons in the TCA cycle, the low overlap between their protein interaction data and our CN-PAGE approach indicates that PPIs and metabolon formation in the TCA cycle is not yet fully resolved. Metabolons are defined as interactions of sequential enzymes of a metabolic pathway that allow efficient channelling of metabolites^26,28,30^. We therefore analysed the 74 PPIs predicted by ROC curve analysis for interactions between sequential enzymes of the TCA cycle which have not been reported by Zhang *et al*. We observed the interaction between the aconitase subunit ACO2 with the isocitrate dehydrogenase subunit IDH6. Furthermore, the two remaining isocitrate dehydrogenase subunits (IDH1 and IDH5) and ketoglutarate dehydrogenase (ODC1-1) were predicted to interact in our dataset. Finally, we predicted the interaction between citrate synthase subunit (CSY4) with ACO1 (Fig. 7D). This specific interaction was not reported by Zhang and co-workers^29^. However, the formation of a metabolon between MDH, CYS and ACO has been described before^31^.

Additionally, our data suggests interactions between known protein complexes, aconitase (ACO) and the three ATP citrate lyase (ACL) subunits, that play an important role in the reductive TCA cycle^32^ (Fig. 7E). While further experiments are required to confirm this putative interaction, it is intriguing to speculate that the formation of a metabolon between ACO and ACL might occur and support the channelling of substrates from isocitrate via citrate to oxaloacetate.

We further hypothesized that the oligomerization state of enzymes may be dependent on environmental factors such as light and temperature. As the TCA cycle operates in different modes in the light and the dark, we used our ED and EN datasets to test this hypothesis^33^. However, we could not observe any change in the oligomerization state of TCA cycle related enzymes between the two time points (Supplemental Table 1). It has been shown that rearrangements in the interaction between TCA cycle complexes plays a role in the response of plant cells to oxidative stress^34^. The absence of significant changes between ED and EN could indicate that under normal growth conditions plants regulated the activity of the TCA cycle enzymes independently of the oligomerisation state – potentially by post translational modifications^35–37^. It is also conceivable that rearrangements in the TCA cycle protein complexes between ED and EN are minor and cannot be resolved with our experimental approach. Moreover, changes in complex composition or abundance between ED and EN could occur mainly in unstable PPIs which are generally underrepresented in our dataset.

In future experiments, the established sample processing and data analysis pipeline could be applied to study changes in protein complex abundance and composition in response to changes in the environment or following the perception of biotic or abiotic stress.

### Quantitative complexome analysis at ED and EN

**Figure 8:**
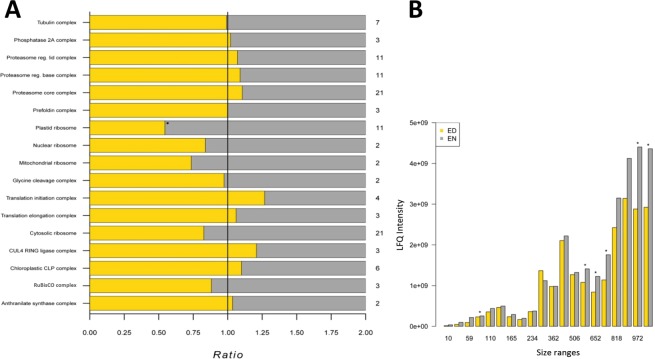
Arabidopsis complexome at the ED and EN. The relative abundances of protein complexes from the GO-Slim database were compared at the ED and EN. Ratios of the AUC from protein complex subunits identified in ED and EN were calculated and averaged for proteins belonging to the same complex. (**A**) The number of proteins the complex is composed of. (**B**) A barplot of the total abundance of ribosomal proteins found in each fraction of the gel. The analysis was performed for all overlapping ribosomal proteins that were present in both experiments.

We further developed our approach to quantitatively compare protein complexes and applied it to study the complexome of *A. thaliana* at the ED and the EN. The high reproducibility of the data allowed us to compare the changes in relative abundance of the protein complexes between the two time points. The area under the curve (AUC) was calculated for all identified proteins, based on the deconvoluted migration profiles. Using the Euclidean distance cut-off obtained by ROC curve analysis (Supplemental Fig. 5), profiles putatively representing proteins of the same protein complex were identified. The data was then filtered for proteins which were present in both, the ED and EN, dataset. This allowed us to specifically investigate changes in abundance at the individual time points and at the same time exclude changes introduced by the protein composition. Next, the mean of the AUC was calculated for each of the protein complexes and compared between the time points. The ratios calculated in this manner are shown as a horizontal barplot (Fig. 8A). A paired t-test was used to test for the significance of observed changes. While most of the protein complexes we identified showed no significant change in abundance between ED and EN, the plastidial ribosomal assemblies showed higher abundances at the EN than at the ED (Fig. 8B). At first this observation seems contradictory to previous studies which showed that the abundance of ribosomal proteins is stable throughout the diurnal cycle in plants^38^. However, it has been shown that the translation activity of ribosomes is higher during the day than during the night^39^. The ribosomal assemblies resolved in our native PAGE analysis have a size of less than 1MD and therefore do not represent fully assembled ribosomes required for translation, but the individual subunits. This can be explained by the molecular weight of the fully assembled plastid ribosome, which is approximately 2 MDa and exceeds the size range of the clear native-PAGE gel used in this study. However, the small subunit has a molecular mass of around 650 kDa and can be resolved in the gel. The large subunit is roughly 1300 kDa and corresponds to the upper limit of the gel size range^40,41^. Consequently, the higher abundance of ribosomal proteins assemblies at EN we observed shows that inactive ribosomes disassemble during the night and their subunits can be resolved in our native PAGE. This explanation also further highlights the ability of our method to accurately detect when changes in the relative protein abundance of protein complexes occur.

## Conclusion

In this report we present new approach for the quantitative analysis of protein complexes. Using CN-PAGE and high-throughput sample preparation for mass spectrometry, we generated dataset to study protein complexes in the model plant *A. thaliana* at the end of the day and the end of the night. Our data analysis pipeline includes the deconvolution of complex protein elution profiles and builds the prediction of protein-protein interactions on the Euclidean distance between profiles in combination with a ROC curve analysis. We show that the established dataset has a high reproducibility and an improved coverage compared to previous studies in the model plant *A. thaliana*. This allows insight into many biological pathways and to prediction of novel putative interactors. What sets our study appart from other approaches for the analysis of protein complexes, is the ability to process samples for biological replicates and treatments in parallel, therby facilitatating quantitative comparison. Taken together, our work is an advance towards the comparative analysis of protein complexes under changing environmental conditions that can be applied to any model organism.

## Methods

### Plant growth and sample preparation

*A. thaliana Col-0* plants were grown on soil in a controlled environment room for 25 days under a short day photoperiod (12 h light/12 h dark, 130 µmol photons m^−2^ s^−1^, 25 °C and 60% ambient humidity). Plant material was harvested into liquid nitrogen at two time points. The first time point was at the end of the 12 hour light period and the second time point was at the end of the 12 hour dark period. Whole rosettes from up to 5 distinct plants were combined to create a biological replicate. A total of four biological replicates were collected at each time point. Plant tissue was ground to a fine powder in liquid nitrogen and proteins were extracted on ice in a native protein extraction buffer (25 mM Tris-Cl, pH 7.0; 20% glycerol [v/v]; 2 × Protease Inhibitor Cocktail (Roche)). Protein estimation was performed using a BCA assay kit (Thermo Scientific) according to the manufacturer’s instruction.

### Native PAGE and in-gel tryptic digestion

Approximately 400 µg of protein from each biological replicate was separated in a large format handcast Tris-glycine gel (16 × 20 cm, gradient 3.5% to 9%) under native conditions using a PROTEAN^®^ II xi cell from Biorad. Following protein separation the gel was stained for 1 hour at room temperature in a Coomassie Blue solution (20% [v/v] methanol, 10% [v/v] acetic acid, and 0.1% [w/v] Coomassie Brilliant Blue R) and then destained for another 2 hours at room temperature in a destaining solution (50% [v/v] methanol and 10% [v/v]). Each lane was fractionated into 20 pieces and the slices were transferred to 96 well plates for the in-gel tryptic digestion procedure^17^. Gel slices were washed three times for 1 hour at 37 °C with a destaining buffer (100 mM ammonium bicarbonate, 50% [v/v] methanol) and subsequently subjected to the in-gel tryptic digest with minor modifications to the sample volumes. The resulting peptides were desalted by reverse-phase chromatography on Finisterre C18 SPE columns (Teknokroma), dried in a vacuum centrifuge and stored at −80 °C.

### Liquid chromatography-mass spectrometry

Prior to analysis peptides were resuspended in 40 µl of resuspension buffer (3% [v/v] acetonitrile, 0.1% [v/v] formic acid). Peptides were separated online by reverse phase liquid chromatography using a NanoLC 1D (Eksigent) before being measured on a LTQ-Orbitrap XL ETD (Thermo Scientific). An in-house made capillary column (75 µm i.d., 8 cm long) was packed with Magic C18 AQ beads (5 µm, 100 Å, Microm) and used for sample loading. Samples were loaded at a flow rate of 0.5 µL min^−1^ in a buffer composed of 3% (v/v) acetonitrile and 0.2% (v/v) formic acid. An acetonitrile concentration gradient (5% to 40% [v/v] acetonitrile) was used to elute peptides over 70 minutes at a flow rate of 0.5 µL min^−1^. The column was then washed for ten minutes with 80% (v/v) acetonitrile at a 0.25 µL min^−1^ flow rate to avoid potential memory effect as it is crucial for quantification study were small number of remaining proteins would have an effect on obtained results. Scan parameters were configured to detect ions in a full scan from 300 to 2000 m/z at a resolution of 35’000. Following detection data dependent tandem mass spectrometry scans were performed for the 7 most abundant ions (AGC target, 500 hits, isolation width m/z, 3, normalized collision energy (NCE), 35%). Dynamic exclusion was applied for 30 secs to peptides for which MS/MS spectra was recorded. MS data was acquired in Proteomics Facility of the ETH Zurich.

### Protein identification and label free quantitation

Raw MS/MS spectra was imported into MaxQuant (version 1.6) for protein identification and quantitation^42^. Peptide identification by the Andromeda search engine was based on the *Arabidopsis thaliana* TAIR10 protein sequence database (35,386 entries)^18^. The following parameters were applied to the analysis: 10 ppm peptide mass tolerance; 0.8 Da MS/MS tolerance; a maximum of two missed cleavages were allowed; a decoy database search with a 1% FDR cutoff on the protein level; carbamidomethylation of cysteine was set as a fixed modification, while the oxidation of methionine was set as variable modification. The “label-free quantification” and “match between runs” settings were also highlighted in the software. Valid peptides were expected to have a minimum length of six amino acids. Peptide quantitation was performed for proteins identified with at least two peptides (a minimum of one unique and one razor) unmodified peptide. Peptides intensity was taken and further normalize by LFQ algorithm. Known contaminants were removed from the analysis. MaxQuant was used to produce the “mean” complexome dataset by combining replicates within the software.

The mass spectrometry proteomics data have been deposited to the ProteomeXchange Consortium via the PRIDE^42^ partner repository with the dataset identifier PXD010919.

### Statistical analysis/Prediction of putative protein complexes/data processing

#### Hierarchical clustering

Single linkage clustering of the data was performed using the in R software. The distance between protein profile peaks were calculated using the Euclidean distance formula in R. Heatmaps were produced in R by applying the hclust function to the data.

#### Deconvolution of protein profiles

The deconvolution procedure starts by identifying the local maxima in a protein profile by applying the turnpoints function from the pastecs package in R. After the local maxima have been defined, each maxima is filtered to retain only the peaks that exceed 20% of the highest relative abundance. To be considered as part of a peak after deconvolution, the signal needs to be at least 5% of the maximum abundance as measured in the total profile. The deconvoluted peaks were obtained by applying a decision tree at each local maxima, which was repeated for each analysed fraction. A deconvoluted peak was considered to terminate when the signal fell below 70% of the maximum relative abundance in the total profile. If this behaviour is not observed the profile is investigated in the next fraction until the signal enters the downward slope and reaches the termination threshold. Alternatively a peak can also be terminated when the relative abundance in a downstream fraction exceeds 70% of the relative relative signal of the previous peak. Peaks obtained by deconvolution are finally crosschecked against the initial local maxima, and where the peaks are in agreement, a verified deconvoluted peak is obtained.

#### Receiver-Operator Characteristics

Receiver-operator characteristics for all experiments were calculated using the GO Slim^19^ annotations for Arabidopsis as a validated set of true interactions. The set of true negative interactions were generated using random combinations of proteins annotated in the database, as well as proteins from the dataset which are not annotated to belong to any protein complex in the GO Slim database. To reduce the risk of accidentally selecting proteins that really interact, only protein pairs that were predicted to have distinct subcellular localizations were accepted in the TN set. The subcellular localization was determined using the SUBA3 database^43^.

#### Complexome comparison

Deconvoluted protein profiles, prepared as previously described, were used for the complexome comparison. Firstly, the GO Slim database^19^ was used a reference to group all of the proteins that belong to the same protein complex. Next, at each of the time points, these groupings were inspected to determine which fraction contained the highest number of coeluting protein peaks. The protein peaks within this fraction were then combined with those of the neighboring fractions (+1/−1) to create a set of all protein peaks representing the protein complex at the investigated size range. Subsequently, the Euclidean distance was calculated between all of the aforementioned profiles within the protein complex. A filtering step was then performed. Only the protein profiles that satisfied the Euclidian distance cutoff of 0.3, as calculated by the ROC curve analysis, were kept. Additionaly, to be retained for the analysis, each of the proteins belonging to the protein complex were expected to have similar profiles in at least three of the biological replicates. Finally, the protein complexes that had the same protein composition at both time points were selected for the complexome comparison. The area under the curve (AUC) was calculated for each of the proteins in the protein complex and the resulting mean values were used for the comparison. A student’s t-test was applied to determine whether changes were statistically significant.

## Supplementary information

Supplementary Dataset 1

## Data Availability

The proteomics datasets produced in this study are available in the PRIDE database using the following accessions: PXD010919.
