# Supplementary material for: Protein Complex Identification and quantitative complexome by CN-PAGE
Source: Sci Rep. 2019 Aug 8;9:11523. doi: 10.1038/s41598-019-47829-7 (PMC6687828; doi:10.1038/s41598-019-47829-7)
Supplement: Supplementary file 1 — Supplementary Dataset 1 [file 41598_2019_47829_MOESM1_ESM.pdf]

Protein Complex Identification and quantitative complexome by CN-PAGE

Michal Gorka[1*], Corné Swart[1], Beata Siemiatkowska[1], Silvia Martínez-Jaime[1], Sebastian Streb[2], Aleksandra Skirycz[1], Alexander Graf[1]

[1] Max Planck Institute of Molecular Plant Physiology, Am Mühlenberg 1, 14476, Golm Germany

[2] Institute for Agricultural Sciences, ETH Zurich, Universitätstrasse 2, 8092 Zürich, Switzerland

*Corresponding author:

Email: gorka@mpimp-golm.mpg.de

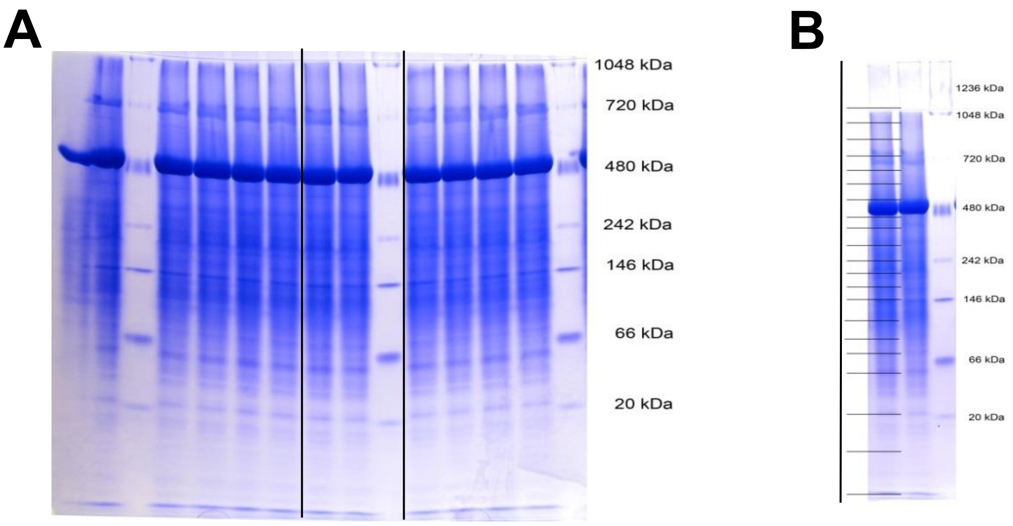

Supplemental Figure 1. **CN-PAGE of native extracts and subsequent fractionation.**

Proteins extracted at the ED and EN were separated by CN-PAGE. A total of four biological replicates were selected for each time-point. (A) Following electrophoresis, the gel was stained with Coomassie Brilliant Blue and a template for fractionation was prepared. The cutting design was shown on a part of a gel (B). Each sample lane was divided into 20 fractions as depicted.

A

B

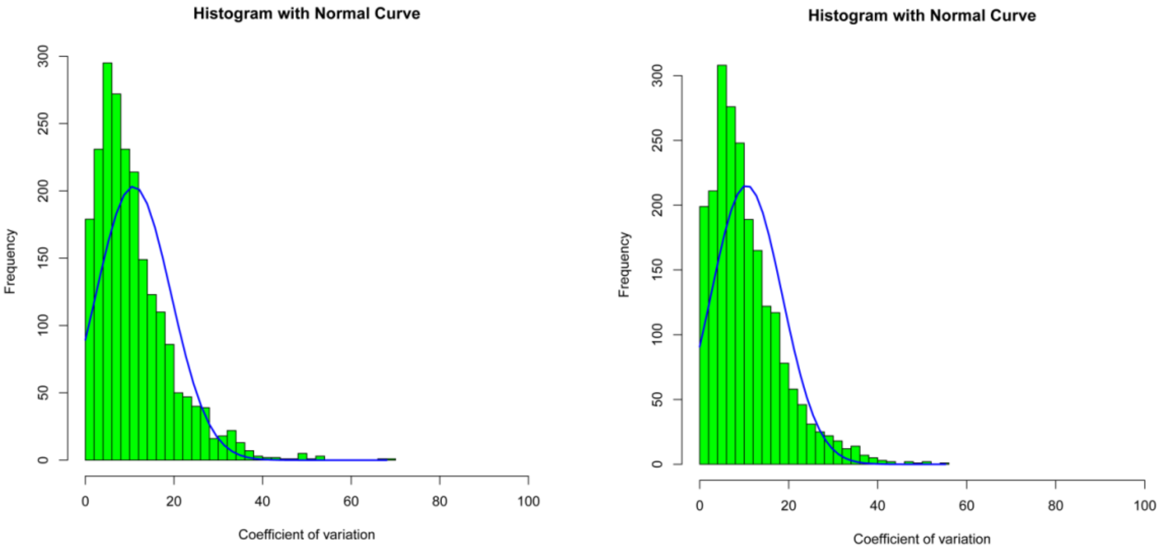

C

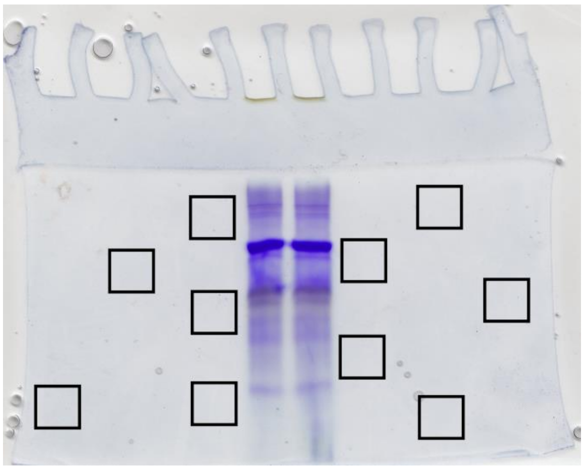

D

AGI code	Protein	Peptides
AT1G11860.3	Glycine cleavage T-protein	1
AT1G16810.2	DHCR7	1
AT1G28430.1	CYP705A24	1
AT1G67090.1	RBCS1A	3
AT1G74160.1	LNG3	1
AT2G30320.1	Pseudouridine synthase	1
AT2G36610.1	ATHB22	1
AT2G42460.2	TRAF-like family protein	1
AT3G10690.1	DNA GYRASE A	1
AT3G22640.1	PAP85	2
AT3G27860.1	PDP6	1
AT4G04180.1	P-loop_NTPase family	1
AT4G18980.1	AtS40-3	1
AT4G28520.2	cruciferin 3	1
AT4G30960.1	CIPK6	1
AT5G14011.1	unknown protein	1
AT5G38430.1	RBCS1B	3
AT5G44120.1	CRA1	2
ATCG00480.1	ATPB	1
ATCG00490.1	RBCL	11

Supplemental Figure 2. Reproducibility of the native-PAGE experiments.

A coefficient of variation analysis was performed to determine the technical variation in the data obtained from the four biological replicates for the (A) ED (B) EN. Histograms with normal curves were used to visually present the results. (C) Ten fractions were randomly excised from native-PAGE gel, processed and analyzed via mass spectrometry. (D) Table contains all identified proteins in 10 gel slices by LC-MS/MS.

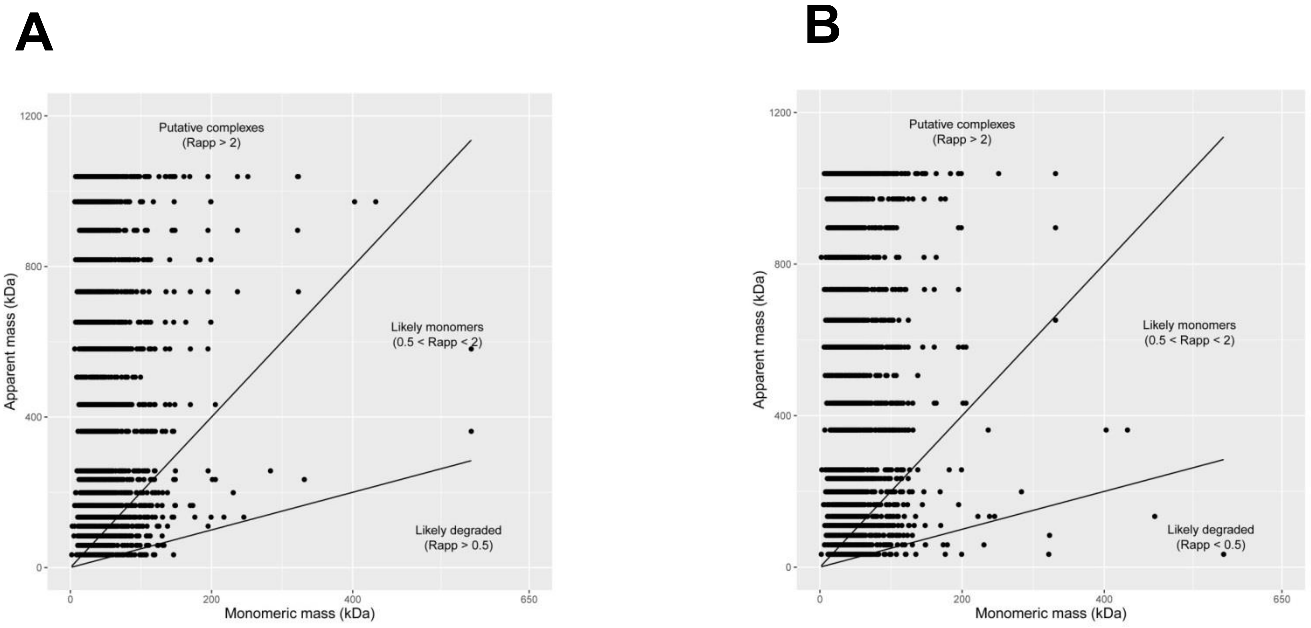

Supplemental Figure 3. Oligomerization state of proteins.

Scatterplots showing the distribution of the apparent mass of proteins identified at the (A) ED and EN (B). Circles represent the apparent mass (y-axis) of proteins as experimentally determined, in comparison to their monomeric masses (x-axis) obtained from the TAIR10 database.

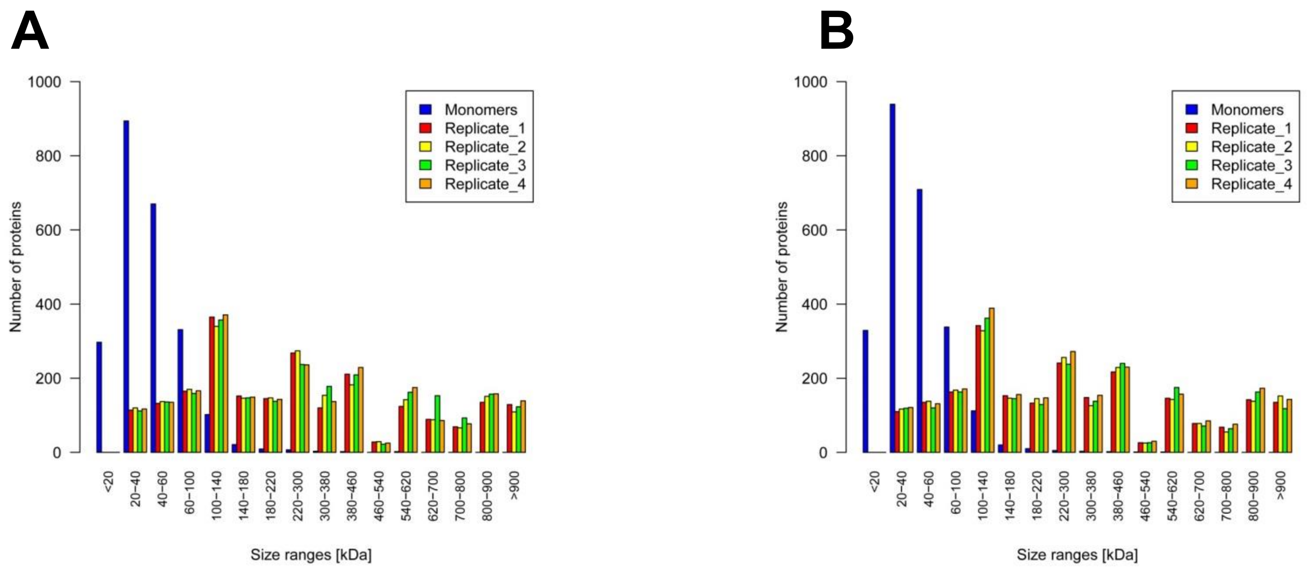

Supplemental Figure 4. Distribution of monomeric and experimentally determined apparent masses.

The histogram shows the distributions of all the identified proteins within the CN-PAGE experiments. The monomeric masses and the experimentally determined masses are represented for (A) ED and (B) EN. All four biological replicates are investigated at each of the time-points.

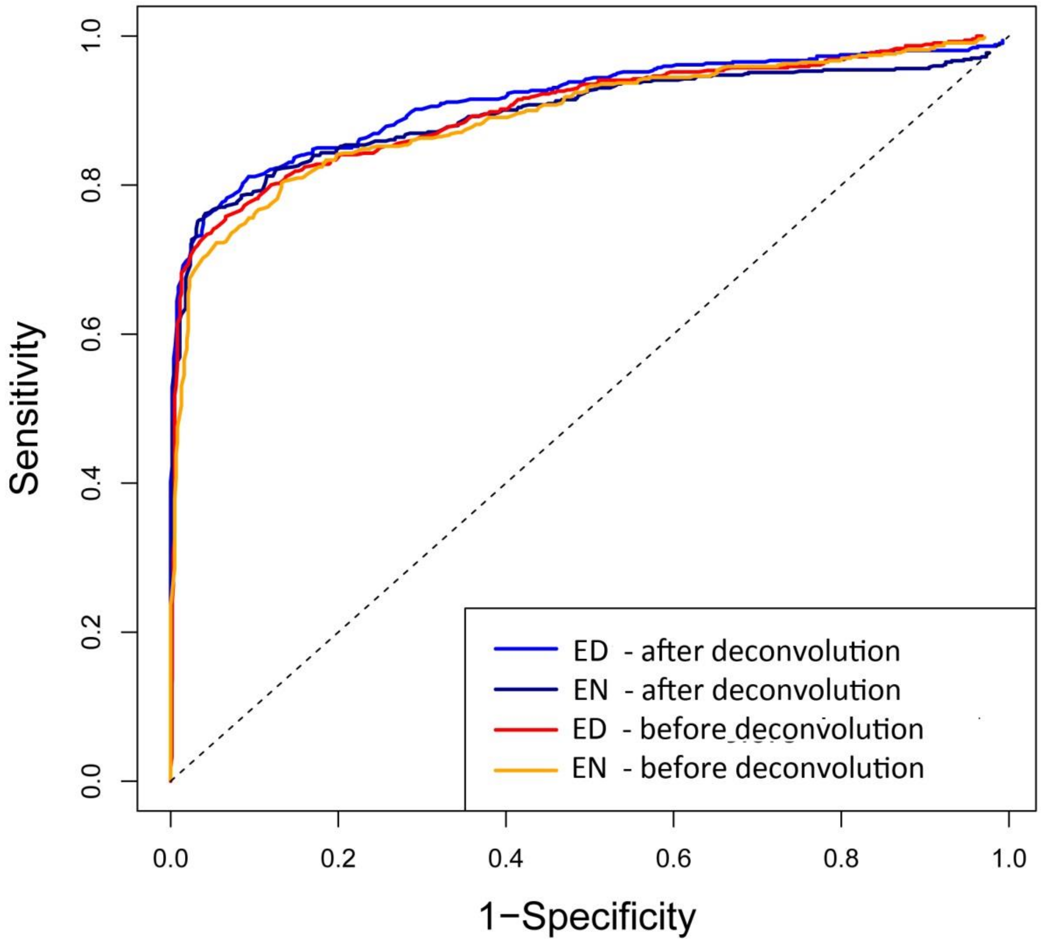

Supplemental Figure 5. Receiver Operating Characteristic curves for the ED and EN.

ROC curves to support the validation of protein-protein interactions were calculated for the ED and EN. A pair of curves, one based on the original profiles (without deconvolution) and the second on the deconvoluted profiles, were generated for each of the timepoints. The curves were constructed by plotting the TPR (sensitivity) versus FPR (1- specificity) at incrementally increasing threshold settings. The GO Slim database served as a validated set of true interactions, and was used as a reference to verify the observed protein protein interactions. ROC curves using deconvoluted profiles are displayed as blue and dark blue lines for ED and EN, respectively. In contrast curves generated from the original profiles are colored in red for ED and orange for EN.
